# Assessment of the causes and extent of damage to trees of *Olea europaea* subsp. *cuspidata* (Wall. and G.Don) Cif. (wild olive) in the mountains of Oman

**DOI:** 10.1371/journal.pone.0343218

**Published:** 2026-03-05

**Authors:** Thuraiya Al Jabri, Alastair Culham, Richard H. Ellis

**Affiliations:** 1 School of Biological Sciences, University of Reading, Reading, United Kingdom; 2 School of Agriculture, Policy and Development, University of Reading, Reading, United Kingdom; Sultan Qaboos University College of Science, OMAN

## Abstract

*Olea europaea* subsp. *cuspidata* (Wall. and G.Don) Cif. (wild olive) is one of the key woody species in the mountain habitats of Oman. Wild olive trees are scattered, isolated, and at risk from several threats including climate change, urbanization, browsing, human activity, and the introduction of non-native species. One hundred and eighty-four trees from eight locations in three mountain ranges (Eastern Hajar, Western Hajar, and Dhofar) in Oman were assessed. The extent of damage to trees caused by browsing, drought (dead branches), and human activity (cutting and burning) was scored between 10 June and 5 July 2020. Most olive trees in these mountain ranges exhibited moderate damage, ranging from 21% to 45%, while 29% of wild olive trees experienced high levels of damage, ranging from 45% to 64%. Wild olive trees in the Western Hajar Mountains and Dhofar Mountains showed the greatest damage. Tree height differed significantly among these eight locations across Oman. There was a negative correlation between tree damage and tree height and a positive correlation between tree damage and site slope but no correlation between tree damage and site altitude. No natural regeneration of wild olive was detected in any of the eight locations. Urbanization and over-browsing are putting wild olive at high risk. Action to protect these mountain habitats will be essential to conserve this ecologically important subspecies in these mountains.

## 1. Introduction

*Olea europaea* subsp. *cuspidata* (Wall. and G.Don) Cif. (wild olive), one of the six subspecies of the *Olea europaea* complex [1–3], is adapted to semi-arid and meso-humid climates [4]. It is distributed mainly at mid to high altitudes from South Africa through the Middle East, Pakistan and India to China [1].

Wild olive is common in the northern and southern mountains of Oman and is one of the three dominant trees of the upland ecosystem of the Western Hajar Mountains where most of Oman’s endemic species are found [5]. Being a crop wild relative with potential utility in crop improvement, it has been used as a rootstock for cultivated olive in the region [6]. Our observations in the field suggest that wild olive is of particular significance in Oman’s flora: first as an indicator of habitat quality; second, as a protector of young plants of other species; and third, as a source of food and shelter for a range of native animals. While common and widespread in Oman, there are few places where the trees are in good condition, while most show little or no new growth [7]. Wild olive is listed as of Least Concern (LC) on the National Red List of Oman but is close to being near threatened (NT) [7]. Recently, wild olive in similar habitats was assessed as Vulnerable (VU) in the United Arab Emirates National Red List of Vascular Plants Technical Report [8].

Oman includes part of the Arabian Highlands woodlands and shrublands, one of the 142 terrestrial ecoregions in which effective conservation is required to conserve the world’s most outstanding and representative habitats for biodiversity [9]. About 40% of the world’s land area is made up of arid and semi-arid rangelands, including those in which wild olive occurs, which have a significant impact on the livelihood and well-being of around one-fifth of the human population [10]. The effects of climate change and human activities in recent decades have exacerbated desertification and land degradation in these arid, semi-arid, and dry sub-humid regions [11]. Excessive grazing by livestock has been identified as a primary factor in the degradation of arid and semi-arid land in various areas across the globe [12] resulting in biodiversity loss and decreased land productivity [13,14]. In Oman, wild olive trees are isolated in fragile, vulnerable mountain habitats [5,15,16]. An average tree condition index of only 50% was reported for wild olive trees in Jabal Shams in the Western Hajar Mountains based on foliage cover [17].

Dryland ecosystems are among the most vulnerable to anthropogenic disturbances, including land-use intensification, infrastructure development, and overgrazing, which contribute significantly to the ongoing decline of global plant diversity despite increased conservation efforts [18]. In southern Oman, these pressures are compounded by declining vegetation vigor and increasing land-use intensity in Dhofar, as documented by recent remote-sensing and regional studies linking these changes to reduced monsoonal precipitation and rising temperatures [19–21]. Humans have had a major influence on biodiversity and species distribution at both local and global scales, due to population growth, resource depletion, and technological advancement [22,23]. Land use intensification and other human activities have reduced species distributions worldwide, and approximately 40% of plant species are now threatened with extinction [24].

In Oman, a dryland country, rapid economic growth has altered natural landscapes, especially through the construction of roads and residential buildings [25]. This urbanization process damages natural ecosystems, leading to biodiversity loss. The annual growth of the urban population in Oman reached its highest level on record in 2016 (6.4% p.a.), with 83.6% of the population living in urban areas in 2017 [26]. In response, the Ministry of Agriculture has established land-use regulations to prohibit the conversion of agricultural land to other uses [27]. Nonetheless, urban growth and agricultural expansion have transformed several mountain settlements from rural to urban, with measurable impacts on vegetation patterns in Dhofar [20,26].

As Oman has urbanized, many roads have been constructed to access remote areas, increasing human and livestock access to rare plant sites [25]. Over-browsing by camels, goats, and wild donkeys poses a major threat to the vegetation. *Olea europaea* subsp. *cuspidata* (wild olive) trees are particularly affected, with branches cut and leaves removed for fodder [25]. This has contributed to a notable increase in unpalatable species across Oman’s rangelands [28], further reflecting the ecological impact of these socio-economic changes.

Climate change poses a significant threat to biodiversity worldwide, affecting ecosystems through rising temperatures, altered precipitation patterns, and increased desertification [29]. These changes can disrupt plant morphology, physiology, and life history, ultimately reducing natural regeneration from seeds [30,31].

In the Middle East, countries like Lebanon are already experiencing the adverse effects of climate change. Rising temperatures are expected to negatively impact the development of cultivated olive trees and reduce olive oil production, key components of the region’s agricultural economy [32].

Oman, too, is particularly vulnerable to climate change. The country faces challenges from rising temperatures, unpredictable precipitation, and advancing desertification, all of which pose significant risks to its biodiversity [33]. These changes threaten the delicate balance of Oman’s ecosystems and may hinder the regeneration and survival of native plant species [30,31].

The condition of wild olive trees was assessed at eight locations within each of the three mountain ranges of Oman in this study to cover the natural distribution of this taxon. The objectives were to determine and benchmark the current status of wild olive trees in their natural habitats across Oman, in the Eastern and Western Hajar Mountain ranges in the north and the Dhofar Mountain range in the south; and to identify the extent of damage to these wild trees, and the causes of damage, across these habitats to provide a foundation from which to develop suitable approaches to conservation.

## 2. Materials and methods

**Fig 1:**
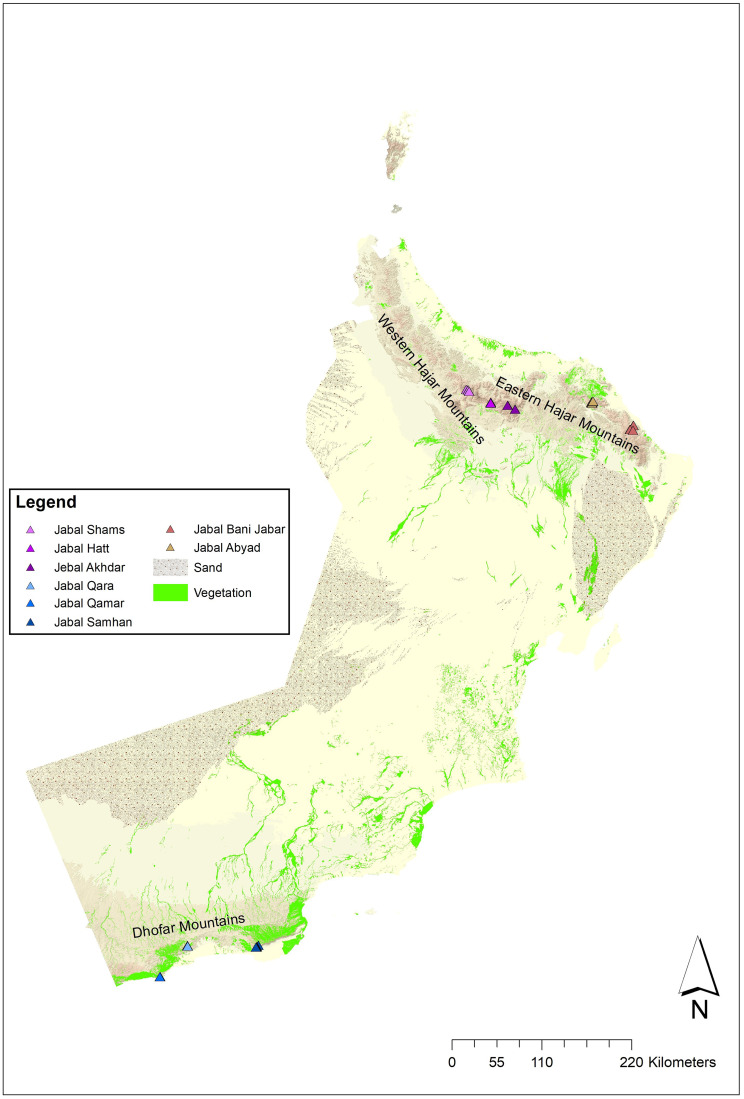
Location of the eight study sites in Oman. Jabal Abyad [EJA] and Jabal Bani Jabir [EJB] are in the Eastern Hajar Mountains in the north of Oman. Jabal Akhdar [WJK], Jabal Hatt [WJH] and Jabal Shams [WJS] are in the Western Hajar Mountains also in the north of Oman. Jabal Samahan [DJS], Jabal Qamar [DJR] and Jabal Qara [DJQ] are in the Dhofar Mountains in the south of Oman. Created by the authors using ArcGIS software and the basemap shapefile from the National Survey and Geospatial Information Authority (NSGIA), Sultanate of Oman (https://www.nsaomangeoportal.gov.om/nsamapviewer/index.html). Basemap republished from NSGIA under a CC BY license, with permission from NSGIA, original copyright 2025.

Wild olive trees were located and assessed at eight locations in the three mountain ranges of Oman: (1) Western Hajar Mountains: Jabal Akhdar (WJK), Jabal Hatt (WJH) and Jabal Shams (WJS); (2) Eastern Hajar Mountains: Jabal Abyad (EJA) and Jabal Bani Jabir (EJB); (3) Dhofar Mountains: Jabal Samahan (DJS), Jabal Qamar (DJR) and Jabal Qara (DJQ) (Fig 1). The Jabal Abyad (EJA) location in the Eastern Hajar Mountain included the nature reserve of Wadi Sareen for which permission was obtained from the Office of Conservation of the Environment (17/SN 2020).

A total of 184 trees were evaluated between 10 June and 5 July 2020. Twenty trees were selected for study at each location, except for Jabal Abyad (EJA), where 44 trees were assessed. The latter was to compensate for the one fewer study site in the Eastern Hajar Mountains so that each mountain range provided a similar total number of trees. The wild olive trees were often isolated, or in small clumps of two or three, and occasionally four or five, trees. The distance between trees assessed within each site ranged from 6 m up to 4 km. Due to the mountainous terrain, the transects to locate and select trees for assessment were approximately linear. The distance between trees in the survey area varied significantly among the eight locations due to variation in topography and terrain. The altitude and coordinates of each tree were determined with a hand-held GPS receiver (GPSmap 62s GARMIN, Southampton, UK) and the slope was extracted from the 1 km Digital Elevation Model (DEM) using ArcMap 10.5.1 (ArcGIS 10.5.1, ESRI, California, USA).

### 2.1. Damage to wild olive trees

**Fig 2:**
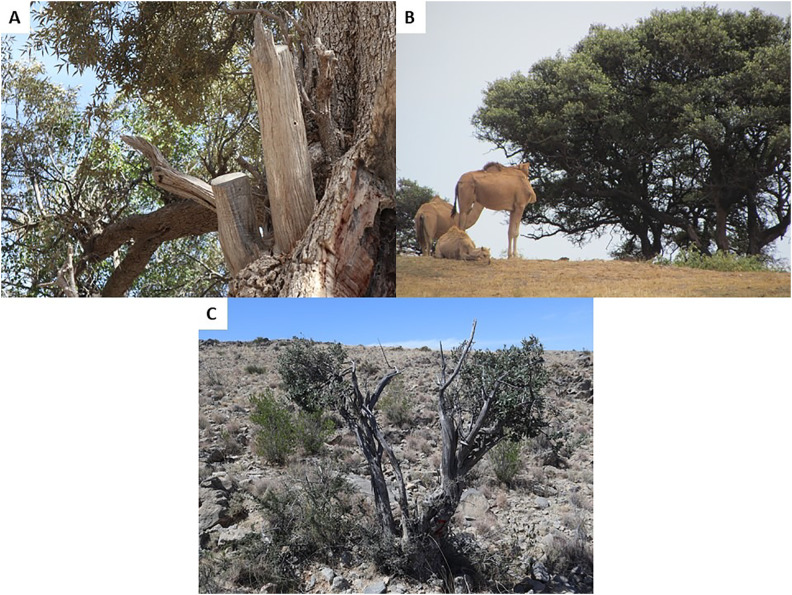
Types of damage to wild olive trees. A, cut branches; B, browsing; C, dead branches. Photographs taken by the authors during fieldwork in Oman.

**Fig 3:**
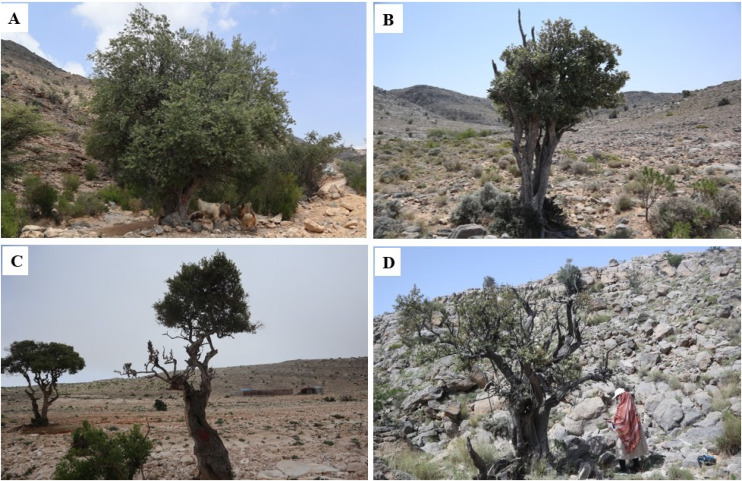
Scoring of damage to wild olive trees in Oman. A, damage score 0 (0-20% of tree damaged), B score 1 (21-45%), C, score 2 (46-64%), D score 3 (>65%). Photographs taken by the authors during fieldwork in Oman.

Damage to each tree was scored visually and the percentage of branches damaged (dead, cut, or browsed) recorded. This continuous data was also categorised on a four-point scale (Figs 2 and 3), ranging from 0 for low damage (i.e., 0–20% damaged branches), 1 for moderate damage (i.e., 21–45%), 2 for high damage (46–64%) and 3 indicating severe damage (i.e., ≥ 65%).

### 2.2. Tree height

The height (*H*, m) of each tree assessed for damage was measured using a clinometer (Steren 203–661, Taiwan) and estimated from the clinometer reading (CR, %) by *H* = (*CR*/100 × *DT*)+ *DCG* where DT = distance from clinometer to tree (m), and DCG = distance of clinometer from ground (m).

### 2.3. Data analysis

The observations for tree damage scores were calculated as frequencies, with means and standard deviation for each location. Tree damage among and within mountain ranges was analysed using Kruskal-Wallis one-way analysis of variance (ANOVA) and pairwise comparison made using Dunn’s test*.* Observations for tree height amongst and within the three mountain ranges were subjected to ANOVA and means separation performed using Tukey’s test. The strength of associations between tree damage and other variables (tree height, site altitude and slope) were computed by Pearson correlation coefficients from all observations (n = 184 trees). Data were analysed in R 4.0.4 [34].

## 3. Results

### 3.1. Damage to wild olive trees

Considerable damage to the wild olive trees (Figs 2 and 3) was detected at all eight locations across the three mountain ranges studied (Table 1). Overall, only 5% of trees were scored in the lowest category of damage, just over half showed moderate damage, whilst 42% showed high or severe damage. The scale of damage did not differ among the three mountain ranges on average, with means varying only between 36.7 and 37.9% damaged branches (Table 2). However, tree damage differed significantly amongst locations across these mountain ranges (Table 2). Trees at the five sites (Jabal Qamar [DJR], Jabal Qara [DJQ], Jabal Abyad [EJA], Jabal Akhdar [WJK], Jabal Shams [WJS]) were less damaged than those at the remaining three sites (Jabal Samhan [DJS], Jabal Bani Jabir [EJB], and Jabal Hatt [WJH]).

**Table 1:** Scoring of wild olive tree damage at eight locations across three mountain ranges in Oman.

Damage score (%)^a^	Number of trees in each category^b^								Mean (%)
	South/ Dhofar mountains			East/ Eastern Hajar mountains		West/ Western Hajar mountains			
	DJQ	DJR	DJS	EJA	EJB	WJH	WJK	WJS	
0 (0-20%)	0	0	0	1	0	0	5	3	5
1 (21-45%)	15	15	6	30	8	3	11	10	53
2 (46-64%)	4	5	8	12	7	8	4	6	29
3 (≥65%)	1	0	6	1	5	9	0	1	13

^a^Percentage of branches damaged on each tree.

^b^Twenty trees were assessed for each population, except EJA where 44 trees were assessed (see Table 2).

**Table 2:** Wild olive trees assessed (n), mean tree height (m), mean percentage of damaged branches (%), with mean altitude and mean slope of the site of trees at eight locations in the mountains of Oman.

Mountains	n	Tree height (m, ± s.d.)^a^	Mean damage (%, ± s.d.)^a^	Median damage (%)	Altitude (m)	Slope (%)
Dhofar (D)/Southern Oman						
Jabal Qamar (DJR)	20	6.21 (±1.57) a	31.2 (±11.1) abc	25	826	12.8
Jabal Qara (DJQ)	20	6.45 (±1.24) a	32.5 (±14.0) abc	25	965	20.0
Jabal Samhan (DJS)	20	3.40 (±1.24) c	50 (±19.9) ad	50	1314	34.7
Mean (D)		5.35 (±1.93)	37.9 (±17.5)	25	1036	18.2
Eastern Hajar (EH)						
Jabal Bani Jabir (EJB)	20	4.17 (±1.14) bc	46.2 (±20.3) acd	50	1525	14.0
Jabal Abyad (EJA)	44	4.79 (±1.08) b	32.4 (±13.8) bc	25	1792	12.8
Mean (EH)		4.60 (±1.13)	36.7 (±17.2)	25	1708	13.2
Western Hajar (WH)						
Jabal Hatt (WJH)	20	4.12 (±1.04) bc	57.5 (±18.3) d	50	1932	13.5
Jabal Akhdar (WJK)	20	6.81 (±1.31) a	23.8 (±17.2) b	25	2011	8.3
Jabal Shams (WJS)	20	7.06 (±2.47) a	31.2 (±19.7) abc	25	2098	8.2
Mean (WH)		6.00 (±2.16)	37.5 (±23.2)	25	2014	9.9

^a^Different letters indicate a significant difference (*p* < 0.05) in tree height or damage between locations.

On average, trees in Jabal Akhdar (WJK) were least damaged (23.8% of branches) and those in Jabal Hatt (WJH) had the greatest damage (57.5%, Table 2); both locations are in the Western Hajar Mountains.

Whilst fruit production was observed, no wild olive seedlings or saplings were detected during the four-week expedition.

### 3.2. Tree height, altitude and slope

The tallest trees were recorded in the Western Hajar Mountains (6.0 m), and the shortest (4.6 m) in the Eastern Hajar Mountains (Table 2). There was no significant variation in tree height between the two Eastern Hajar sites. In contrast, clear differences were observed among locations within both the Dhofar and Western Hajar mountain ranges (Table 2). Trees in Jabal Shams were the tallest on average (7.06 m), while those in Jabal Samhan were the shortest (3.40 m). The maximum recorded height was 12.34 m in Jabal Shams, whereas the smallest individual (1.4 m) was found in Jabal Samhan (S1 Table).

The mean altitudes of the wild olive trees assessed ranged from 826 m (Jabal Qamar) to 2098 m (Jabal Shams). Those in the Dhofar Mountains were growing at the lowest altitudes on the steepest slopes, while those in the Western Hajar Mountains were growing at the highest altitudes on the shallowest slopes (Table 2). The extent of damage to the 184 trees was correlated negatively with tree height and positively with the slope at the tree’s site, but not with the site’s altitude (Table 3).

**Table 3:** Associations among biophysical and stand structure parameters of wild olive trees across eight locations in the mountains of Oman (n = 184), based on Pearson correlation coefficients.

	Tree damage	Tree height	Altitude
Tree height	−0.46***		
Altitude	−0.03^NS^	0.01^NS^	
Slope	0.27***	−0.39***	−0.16*

*p < 0.05, ***p < 0.001, ^NS^ = not significant (*p* > 0.05).

## 4. Discussion

### 4.1. Damage to wild olive trees

The Hajar mountains of Oman are a local centre for plant endemism within the Arabian Peninsula [35,36]. Over-browsing is a considerable threat to wild olive in the northern mountains of Oman with goats, camels, and feral donkeys consuming fresh leaves of *Olea* and other species [25]. Damage to wild olive trees from over-browsing is confirmed by the current study and shown to occur in all three mountain areas of Oman. Other causes of damage detected include cutting for firewood and fodder and trunk burning. The damage was considerable, with 95% of the wild olive trees assessed showing moderate, high, or severe damage, and widespread at all eight locations across the three mountain ranges of Oman (Table 1).

Despite fruit production being observed in all eight locations, natural regeneration of wild olive was not detected, confirming an earlier report of no rejuvenation [7]. Hence the failure to regenerate is caused by problems occurring between seed production and seedling establishment. The failure of wild olive to regenerate naturally may be a consequence of high damage by livestock [37], but the extreme climate and its variability might also be a factor [38,39].

Amongst the eight locations studied, populations of Jabal Akhdar (WJK) in the Western Hajar Mountains showed the least damage (Table 2), despite substantial recent urbanization. However, the direct loss of habitat from urbanization was not included in these assessments; only extant trees were studied. Populations of Jabal Hatt (WJH), also in the Western Hajar Mountains, showed the greatest damage (Tables 1 and 2). The three locations studied in the Western Hajar Mountains showed greater variation in mean damage percentage (23.8% – 57.5%) than those within the other mountain ranges (Table 2). Jabal Hatt is located between the capital Muscat and the Al Batinah Governorate, and the area is easy to access due to less difficult road construction through this terrain which is less mountainous than either Jabal Akhdar (WJK) or Jabal Shams (WJS).

Damage to trees from anthropogenic activities was particularly apparent in Jabal Hatt and Jabal Akhdar with many burnt tree trunks observed. Greater human activity has been reported in Jabal Akhdar than in Jabal Shams [17]. However, Jabal Hatt showed the greatest damage to trees and Jabal Akhdar the least of all eight locations (Table 2). Hence, although evidence of anthropogenic activity was visible in Jabal Akhdar, with branches of wild olive trees used by tourists to fuel barbecues and campfires, the comparatively low damage to wild olive trees overall at this location suggests other causes of damage are of greater concern nationally.

Anthropogenic activities also damaged wild olive habitats in the Dhofar Mountains. This mountain range is subject to the southwest monsoon from mid-June to mid-September which forms dense fogs and supports tropical woodlands [25]. Many visitors travel to the Dhofar Mountains during this period, which may disturb habitats. About 13% of wild olive trees here showed 65% or more branches damaged (from both human activities and over-browsing, Table 1) and mean damage ranged from 31.2–50% amongst the three locations (Table 2). Pastoralism remains strong in this area and the number of cattle has expanded greatly since the 1970s [40,41]. The tribal pastoralists who herd their cattle, camels, and goats in these mountains have been supported to increase livestock numbers through the provision of access roads, freshwater wells, subsidized livestock feed, and veterinary services [25]. This has reduced those species palatable to livestock [42,43] with the habitat degraded by these livestock and by rapid development [25]. Over-browsing is a major threat to tree regeneration in arid regions, including our study area. Similar impacts have been reported elsewhere. In Oman, *Juniperus excelsa* woodlands show poor regeneration due to livestock grazing [44]. In Ethiopia, heavy browsing has limited the recovery of native species [45]. In Egypt, overgrazing by camels and goats affects key desert trees such as *Acacia tortilis* [46].

One of the Eastern Hajar mountain locations studied was the Wadi Sareen nature reserve in Jabal Abyad. Wild olive trees there showed the fourth lowest level of damage and significantly less than the two most damaged sites (Table 2). Hence the protection within the nature reserve may be reducing damage by visitors and livestock. Nonetheless, natural regeneration of wild olive was not observed in the reserve, despite the Hajar Mountains spanning steppe zones (BSh, BSk) with sufficient precipitation to support seedlings [47]. The absence of seedlings in both Hajar and Dhofar populations suggests multiple bottlenecks beyond climate, including low germination, microsite limitations, and herbivory by livestock and feral animals [21,48]. Experimental exclosures and planting in moist or sheltered microsites have been shown to improve seedling establishment in Afromontane and semi-arid systems [49–51], indicating that similar approaches could enhance wild olive recruitment in Oman.

### 4.2. Tree height, altitude and slope

Mean tree height in the eight locations across the three mountain ranges ranged from 3.40 to 7.06 m (Table 2). Tree damage was greater the shorter the tree (Table 3). Anthropogenic activity can affect tree height. For example, it reduced the mean height of 193 tree species in Atewa Range Forest Reserve, Ghana [52] and in five species of trees of *Polylepis* in Peru [53]. The negative correlation between damage to the trees and their height is compatible with two opinions; shorter trees might be more susceptible to damage (e.g., from browsing), or trees might be shorter because they have been damaged more. Both are likely and are not mutually exclusive. This correlation, the shorter the tree the greater the damage, accords with the absence of wild olive seedlings (i.e., no regeneration).

Altitude was not associated with damage to, or the height of, wild olive trees (Table 3) with least damage detected at both low (Jabal Qamar, 826 m) and high (Jabal Akhdar, 2011 m) locations (Table 2). Most trees in the Western Hajar Mountains were growing on plateaus or wadis (valleys), whereas in the Eastern Hajar Mountains most were on steeper slopes (S1 Table). The correlation of damage with slope (Table 3) might be due to shallower soil on steep sites with lower nutrient and water availability which in turn affect tree height [54–57], as does more rapid run off of rain [58,59] and the root flexing on steep slopes caused by wind [60]. Slope, tree height and damage were all correlated (Table 3) and it is pertinent to note that, on average, the wild olive trees at Jabal Samhan were the shortest, grew on the steepest slopes, and showed the greatest damage (Table 2).

### 4.3. Conservation perspective

The rate at which wild olive trees are being lost in the mountains of Oman is not known, but the failure to find any evidence of natural regeneration in any location, and the evidence of damage to the majority of trees at all eight locations, suggests that action to conserve wild olive is urgently required throughout the mountains of Oman. This is necessary not only for the conservation of this crop wild relative but also for the important role it plays in the ecosystems of these mountain habitats [5]. The present results suggest the status of wild olive on the National Red List of Oman, Least Concern but close to being near threatened [7], should now be reevaluated.

A strategy to conserve wild olive in the mountains of Oman should protect existing trees from further damage and support its natural regeneration (by germinating seeds *ex situ* from collected fruits and then replanting sapling trees at the original sites and/or *in situ* by protecting the native habitat until young seedlings are well established). Clearly, long-term protection of seedlings from grazing and trees from browsing and cutting would be required to enable tree establishment and growth at these sites. Different approaches might be required for different sites across Oman. Protection of existing wild olive trees is easier in some locations, such as the nature reserve located in Jabal Abyad (EJA), whereas a proactive planting policy may be necessary in poorer sites such as Jabal Hatt (WJH), Jabal Samhan (DJS), and Jabal Bani Jabir (EJB) in the Western Hajar, Dhofar, and Eastern Hajar Mountains. Further action to conserve wild olive in Oman should include the establishment of a gene bank to ensure long-term availability of its genetic resources.

## 5. Conclusions

Our baseline survey shows that the future of wild olive, an integral component of the mountain habitats of Oman, is at risk. No regeneration of wild olive was observed in any of the three mountain ranges of Oman, whilst almost all trees surveyed were damaged by over-browsing and/or human activities. A plan is now required to conserve wild olive trees *in situ* to protect ecosystems across these mountain habitats from the north (Western and Eastern Hajar Mountains) and south of Oman (Dhofar Mountains), thereby benefiting a wide range of flora and fauna.

This study highlights the lack of baseline data for comparative analyses and provides an initial dataset to address this gap. However, more comprehensive studies conducted over longer time periods and supported by vehicles capable of accessing remote sites are required. Future research should incorporate finer-resolution topographic data, cause-specific damage assessments, spatially explicit analyses that account for site-level effects, and clearly defined regeneration survey protocols. The integration of UAV- or satellite-based imagery could further improve the objectivity, spatial coverage, and repeatability of damage assessments. In addition, long-term population monitoring, and comprehensive population genetic studies are recommended.

## Supporting information

S1 Table Information on the 184 individual wild olive trees assessed at eight locations across the three mountain ranges of Oman. (DOCX)
