# Supplementary material for: Assessment of the causes and extent of damage to trees of Olea europaea subsp. cuspidata (Wall. and G.Don) Cif. (wild olive) in the mountains of Oman
Source: PLoS One. 2026 Mar 5;21(3):e0343218. doi: 10.1371/journal.pone.0343218 (PMC12962520; doi:10.1371/journal.pone.0343218)
Supplement: S1 Table — (DOCX) [file pone.0343218.s001.docx]

**Table S1** Information on the 184 individual wild olive trees assessed at eight locations across the three mountain ranges of Oman.

| Mountain Range | Jabal | Tree | Topology Type | Tree Height (m) | Altitude (m) | Tree damage score | Slope |
| --- | --- | --- | --- | --- | --- | --- | --- |
| Eastern Hajar | EJA | 1 | Wadi | 5.14 | 1720 | 1 | 3.161224 |
| Eastern Hajar | EJA | 2 | Wadi | 3.94 | 1704 | 1 | 8.43886 |
| Eastern Hajar | EJA | 3 | Wadi | 4.54 | 1714 | 1 | 2.012496 |
| Eastern Hajar | EJA | 4 | Wadi | 5.94 | 1723 | 1 | 5.493638 |
| Eastern Hajar | EJA | 5 | Plateau | 2.92 | 1791 | 1 | 2.608361 |
| Eastern Hajar | EJA | 6 | Wadi | 4.84 | 1787 | 1 | 9.644829 |
| Eastern Hajar | EJA | 7 | Wadi | 4.54 | 1788 | 2 | 6.379008 |
| Eastern Hajar | EJA | 8 | Wadi | 6.4 | 1783 | 1 | 22.66165 |
| Eastern Hajar | EJA | 9 | Wadi | 5.62 | 1786 | 1 | 14.47583 |
| Eastern Hajar | EJA | 10 | Wadi | 4.54 | 1786 | 2 | 11.53906 |
| Eastern Hajar | EJA | 11 | Wadi | 4.18 | 1794 | 1 | 4.709958 |
| Eastern Hajar | EJA | 12 | Wadi | 5.39 | 1794 | 2 | 14.82186 |
| Eastern Hajar | EJA | 13 | Wadi | 5.74 | 1796 | 1 | 6.147904 |
| Eastern Hajar | EJA | 14 | Slope | 4.84 | 1802 | 1 | 2.409807 |
| Eastern Hajar | EJA | 15 | Wadi | 6.28 | 1799 | 1 | 2.55877 |
| Eastern Hajar | EJA | 16 | Wadi | 5.32 | 1802 | 2 | 4.412967 |
| Eastern Hajar | EJA | 17 | Wadi | 4.18 | 1811 | 2 | 7.71922 |
| Eastern Hajar | EJA | 18 | Wadi | 6.02 | 1814 | 1 | 13.90638 |
| Eastern Hajar | EJA | 19 | Wadi | 4.48 | 1816 | 1 | 8.654594 |
| Eastern Hajar | EJA | 20 | Wadi | 4.54 | 1816 | 3 | 5.7942 |
| Eastern Hajar | EJA | 21 | Wadi | 4.04 | 1755 | 1 | 8.572497 |
| Eastern Hajar | EJA | 22 | Wadi | 4.78 | 1771 | 1 | 19.20598 |
| Eastern Hajar | EJA | 23 | Wadi | 4.13 | 1797 | 2 | 15.41911 |
| Eastern Hajar | EJA | 24 | Wadi | 4.48 | 1811 | 2 | 7.71922 |
| Eastern Hajar | EJA | 25 | Wadi | 6.44 | 1834 | 0 | 1.443028 |
| Eastern Hajar | EJA | 26 | Wadi | 4.84 | 1840 | 1 | 2.981721 |
| Eastern Hajar | EJA | 27 | Wadi | 3.52 | 1846 | 1 | 5.891434 |
| Eastern Hajar | EJA | 28 | Wadi | 4.54 | 1846 | 1 | 10.47887 |
| Eastern Hajar | EJA | 29 | Wadi | 5.23 | 1758 | 2 | 3.677042 |
| Eastern Hajar | EJA | 30 | Wadi | 4.44 | 1764 | 1 | 2.417152 |
| Eastern Hajar | EJA | 31 | Slope | 3.34 | 1870 | 2 | 11.59972 |
| Eastern Hajar | EJA | 32 | Slope | 2.62 | 1861 | 2 | 13.4814 |
| Eastern Hajar | EJA | 33 | Slope | 3.34 | 1833 | 1 | 18.79305 |
| Eastern Hajar | EJA | 34 | Slope | 3.24 | 1836 | 1 | 14.69304 |
| Eastern Hajar | EJA | 35 | Wadi | 4.27 | 1837 | 2 | 5.517844 |
| Eastern Hajar | EJA | 36 | Wadi | 4.24 | 1836 | 1 | 7.680407 |
| Eastern Hajar | EJA | 37 | Wadi | 4.34 | 1829 | 1 | 5.209476 |
| Eastern Hajar | EJA | 38 | Slope | 5.44 | 1787 | 1 | 29.16681 |
| Eastern Hajar | EJA | 39 | Slope | 3.28 | 1797 | 1 | 25.24041 |
| Eastern Hajar | EJA | 40 | Slope | 6.49 | 1767 | 1 | 38.88688 |
| Eastern Hajar | EJA | 41 | Slope | 5.46 | 1759 | 2 | 38.05655 |
| Eastern Hajar | EJA | 42 | Wadi | 7.57 | 1724 | 1 | 21.31059 |
| Eastern Hajar | EJA | 43 | Slope | 4.9 | 1724 | 1 | 50.76096 |
| Eastern Hajar | EJA | 44 | Slope | 6.58 | 1718 | 1 | 48.86974 |
| Eastern Hajar | EJB | 1 | Slope | 4.84 | 1541 | 2 | 22.13206 |
| Eastern Hajar | EJB | 2 | Slope | 4.3 | 1538 | 1 | 20.3144 |
| Eastern Hajar | EJB | 3 | Slope | 2.44 | 1516 | 1 | 17.01916 |
| Eastern Hajar | EJB | 4 | Wadi | 2.38 | 1517 | 2 | 16.07908 |
| Eastern Hajar | EJB | 5 | Wadi | 3.64 | 1505 | 2 | 15.88252 |
| Eastern Hajar | EJB | 6 | Slope | 3.94 | 1509 | 3 | 49.87972 |
| Eastern Hajar | EJB | 7 | Plateau | 4.3 | 1612 | 3 | 3.878279 |
| Eastern Hajar | EJB | 8 | Plateau | 5.14 | 1618 | 2 | 5.452968 |
| Eastern Hajar | EJB | 9 | Wadi | 3.34 | 1619 | 2 | 15.3539 |
| Eastern Hajar | EJB | 10 | Wadi | 3.94 | 1606 | 3 | 3.543479 |
| Eastern Hajar | EJB | 11 | Wadi | 4.66 | 1601 | 1 | 4.379995 |
| Eastern Hajar | EJB | 12 | Wadi | 3.88 | 1603 | 2 | 9.344626 |
| Eastern Hajar | EJB | 13 | Wadi | 5.68 | 1603 | 1 | 16.01158 |
| Eastern Hajar | EJB | 14 | Wadi | 4.74 | 1599 | 1 | 10.4066 |
| Eastern Hajar | EJB | 15 | Wadi | 6.04 | 1599 | 2 | 20.9165 |
| Eastern Hajar | EJB | 16 | Wadi | 5.1 | 1597 | 1 | 4.406694 |
| Eastern Hajar | EJB | 17 | Wadi | 5.53 | 1330 | 1 | 10.42529 |
| Eastern Hajar | EJB | 18 | Wadi | 4.78 | 1331 | 1 | 2.655642 |
| Eastern Hajar | EJB | 19 | Wadi | 2.18 | 1325 | 3 | 23.13836 |
| Eastern Hajar | EJB | 20 | Wadi | 2.56 | 1324 | 3 | 8.944682 |
| Western Hajar | WJH | 1 | Plateau | 4.84 | 1964 | 1 | 6.911957 |
| Western Hajar | WJH | 2 | Plateau | 4.44 | 1964 | 2 | 9.078011 |
| Western Hajar | WJH | 3 | Plateau | 3.76 | 1970 | 2 | 7.212235 |
| Western Hajar | WJH | 4 | Plateau | 4.36 | 1973 | 3 | 12.32827 |
| Western Hajar | WJH | 5 | Plateau | 3.76 | 1975 | 2 | 12.06319 |
| Western Hajar | WJH | 6 | Plateau | 3.88 | 1962 | 2 | 2.756929 |
| Western Hajar | WJH | 7 | Plateau | 3.94 | 1951 | 3 | 4.596785 |
| Western Hajar | WJH | 8 | Plateau | 3.57 | 1949 | 3 | 10.48284 |
| Western Hajar | WJH | 9 | Plateau | 4.41 | 1947 | 1 | 7.613307 |
| Western Hajar | WJH | 10 | Wadi | 7.04 | 1946 | 2 | 2.987739 |
| Western Hajar | WJH | 11 | Plateau | 4.02 | 1926 | 1 | 9.714892 |
| Western Hajar | WJH | 12 | Plateau | 3.88 | 1921 | 2 | 9.536821 |
| Western Hajar | WJH | 13 | Slope | 2.92 | 1926 | 3 | 10.17519 |
| Western Hajar | WJH | 14 | Slope | 2.8 | 1916 | 2 | 10.39283 |
| Western Hajar | WJH | 15 | Slope | 4.34 | 1897 | 3 | 27.81609 |
| Western Hajar | WJH | 16 | Slope | 6.34 | 1889 | 3 | 24.88242 |
| Western Hajar | WJH | 17 | Wadi | 4.06 | 1885 | 3 | 17.10379 |
| Western Hajar | WJH | 18 | Slope | 2.86 | 1890 | 3 | 27.47443 |
| Western Hajar | WJH | 19 | Wadi | 3.76 | 1897 | 2 | 19.38126 |
| Western Hajar | WJH | 20 | Wadi | 3.39 | 1898 | 3 | 36.50825 |
| Western Hajar | WJK | 1 | Wadi | 7.64 | 1908 | 2 | 7.103393 |
| Western Hajar | WJK | 2 | Wadi | 7.54 | 1906 | 1 | 0.862028 |
| Western Hajar | WJK | 3 | Wadi | 5.95 | 1906 | 2 | 11.65735 |
| Western Hajar | WJK | 4 | Wadi | 7.39 | 1903 | 1 | 1.916342 |
| Western Hajar | WJK | 5 | Wadi | 6.49 | 1902 | 2 | 16.93493 |
| Western Hajar | WJK | 6 | Wadi | 5.95 | 1901 | 1 | 20.31126 |
| Western Hajar | WJK | 7 | Wadi | 6.34 | 1901 | 2 | 21.80638 |
| Western Hajar | WJK | 8 | Wadi | 7.24 | 1901 | 0 | 5.349385 |
| Western Hajar | WJK | 9 | Wadi | 5.04 | 1903 | 1 | 3.632701 |
| Western Hajar | WJK | 10 | Wadi | 8.54 | 1904 | 1 | 4.589091 |
| Western Hajar | WJK | 11 | Wadi | 7.74 | 1902 | 1 | 11.21352 |
| Western Hajar | WJK | 12 | Wadi | 9.82 | 1906 | 0 | 4.486142 |
| Western Hajar | WJK | 13 | Wadi | 5.14 | 1904 | 1 | 21.95872 |
| Western Hajar | WJK | 14 | Wadi | 7.24 | 1906 | 1 | 0.862028 |
| Western Hajar | WJK | 15 | Wadi | 8.04 | 1908 | 1 | 11.23125 |
| Western Hajar | WJK | 16 | Plateau | 5.14 | 2324 | 0 | 3.723182 |
| Western Hajar | WJK | 17 | Plateau | 7.94 | 2335 | 0 | 3.282122 |
| Western Hajar | WJK | 18 | Plateau | 5.04 | 2336 | 0 | 0.645345 |
| Western Hajar | WJK | 19 | Plateau | 6.24 | 2335 | 1 | 0.671579 |
| Western Hajar | WJK | 20 | Wadi | 5.74 | 2334 | 1 | 6.543484 |
| Western Hajar | WJS | 1 | Plateau | 6.34 | 2025 | 1 | 6.316164 |
| Western Hajar | WJS | 2 | Plateau | 7.34 | 2025 | 1 | 6.558771 |
| Western Hajar | WJS | 3 | Plateau | 5.74 | 2029 | 1 | 7.427132 |
| Western Hajar | WJS | 4 | Plateau | 7.64 | 2031 | 2 | 10.9028 |
| Western Hajar | WJS | 5 | Plateau | 5.7 | 2037 | 2 | 10.35874 |
| Western Hajar | WJS | 6 | Plateau | 3.88 | 2037 | 2 | 14.04443 |
| Western Hajar | WJS | 7 | Wadi | 5.54 | 2042 | 1 | 11.18593 |
| Western Hajar | WJS | 8 | Wadi | 4.06 | 2045 | 2 | 15.20318 |
| Western Hajar | WJS | 9 | Wadi | 4.87 | 2053 | 1 | 14.93701 |
| Western Hajar | WJS | 10 | Slope | 6.74 | 2057 | 1 | 26.18994 |
| Western Hajar | WJS | 11 | Wadi | 7.54 | 2086 | 0 | 13.35688 |
| Western Hajar | WJS | 12 | Wadi | 11.74 | 2086 | 0 | 2.335676 |
| Western Hajar | WJS | 13 | Wadi | 12.34 | 2087 | 1 | 4.285998 |
| Western Hajar | WJS | 14 | Plateau | 10.14 | 2186 | 1 | 1.42086 |
| Western Hajar | WJS | 15 | Plateau | 4.74 | 2187 | 2 | 5.429503 |
| Western Hajar | WJS | 16 | Plateau | 5.11 | 2187 | 3 | 7.014936 |
| Western Hajar | WJS | 17 | Plateau | 9.1 | 2191 | 1 | 1.315603 |
| Western Hajar | WJS | 18 | Plateau | 10.24 | 2192 | 1 | 2.331973 |
| Western Hajar | WJS | 19 | Plateau | 6.64 | 2192 | 2 | 1.576052 |
| Western Hajar | WJS | 20 | Plateau | 5.74 | 2192 | 0 | 2.210265 |
| Dhofar | DJQ | 1 | Slope | 8.36 | 965 | 1 | 9.240753 |
| Dhofar | DJQ | 2 | Slope | 7.16 | 966 | 1 | 10.06006 |
| Dhofar | DJQ | 3 | Slope | 5.86 | 965 | 1 | 13.89734 |
| Dhofar | DJQ | 4 | Slope | 6.26 | 970 | 2 | 10.34856 |
| Dhofar | DJQ | 5 | Slope | 5.3 | 966 | 3 | 11.68689 |
| Dhofar | DJQ | 6 | Slope | 4.34 | 960 | 2 | 3.582233 |
| Dhofar | DJQ | 7 | Plateau | 6.05 | 960 | 1 | 10.44136 |
| Dhofar | DJQ | 8 | Plateau | 6.68 | 957 | 1 | 7.42926 |
| Dhofar | DJQ | 9 | Plateau | 6.36 | 959 | 1 | 7.482407 |
| Dhofar | DJQ | 10 | Plateau | 4.96 | 958 | 1 | 5.136112 |
| Dhofar | DJQ | 11 | Plateau | 6.32 | 959 | 1 | 3.982549 |
| Dhofar | DJQ | 12 | Plateau | 5.63 | 960 | 1 | 4.515935 |
| Dhofar | DJQ | 13 | Plateau | 5.36 | 962 | 1 | 2.368202 |
| Dhofar | DJQ | 14 | Plateau | 6.56 | 965 | 2 | 6.028481 |
| Dhofar | DJQ | 15 | Plateau | 5.06 | 964 | 2 | 5.855536 |
| Dhofar | DJQ | 16 | Plateau | 8.62 | 967 | 1 | 4.656673 |
| Dhofar | DJQ | 17 | Plateau | 8.12 | 967 | 1 | 3.384724 |
| Dhofar | DJQ | 18 | Plateau | 5.96 | 974 | 1 | 6.914255 |
| Dhofar | DJQ | 19 | Plateau | 8 | 974 | 1 | 8.228964 |
| Dhofar | DJQ | 20 | Plateau | 8.06 | 975 | 1 | 4.3801 |
| Dhofar | DJS | 1 | Slope | 3.56 | 1317 | 1 | 28.95419 |
| Dhofar | DJS | 2 | Slope | 2.36 | 1317 | 2 | 41.47607 |
| Dhofar | DJS | 3 | Slope | 3.26 | 1310 | 2 | 49.08059 |
| Dhofar | DJS | 4 | Slope | 2.06 | 1312 | 2 | 40.40007 |
| Dhofar | DJS | 5 | Slope | 3.14 | 1307 | 2 | 37.60781 |
| Dhofar | DJS | 6 | Slope | 2.36 | 1310 | 3 | 35.15054 |
| Dhofar | DJS | 7 | Slope | 3.51 | 1310 | 1 | 38.19303 |
| Dhofar | DJS | 8 | Slope | 3.37 | 1308 | 1 | 38.43729 |
| Dhofar | DJS | 9 | Slope | 2.11 | 1309 | 3 | 61.18346 |
| Dhofar | DJS | 10 | Slope | 4.01 | 1310 | 3 | 53.14515 |
| Dhofar | DJS | 11 | Slope | 3.38 | 1309 | 3 | 50.0047 |
| Dhofar | DJS | 12 | Slope | 1.4 | 1311 | 3 | 41.50142 |
| Dhofar | DJS | 13 | Slope | 3.74 | 1304 | 1 | 61.92757 |
| Dhofar | DJS | 14 | Slope | 1.7 | 1304 | 2 | 32.39145 |
| Dhofar | DJS | 15 | Slope | 2.5 | 1308 | 1 | 64.05355 |
| Dhofar | DJS | 16 | Plateau | 5.16 | 1290 | 1 | 5.133143 |
| Dhofar | DJS | 17 | Plateau | 4.88 | 1279 | 2 | 9.979953 |
| Dhofar | DJS | 18 | Wadi | 4.91 | 1365 | 3 | 1.218588 |
| Dhofar | DJS | 19 | Wadi | 4.73 | 1364 | 2 | 2.316499 |
| Dhofar | DJS | 20 | Wadi | 5.86 | 1367 | 2 | 2.43021 |
| Dhofar | DJR | 1 | Plateau | 8.38 | 849 | 1 | 4.349984 |
| Dhofar | DJR | 2 | Plateau | 5.84 | 851 | 1 | 3.199374 |
| Dhofar | DJR | 3 | Slope | 5.38 | 851 | 2 | 16.86986 |
| Dhofar | DJR | 4 | Slope | 6.04 | 853 | 1 | 19.52302 |
| Dhofar | DJR | 5 | Slope | 9.82 | 852 | 1 | 24.05299 |
| Dhofar | DJR | 6 | Slope | 4.34 | 846 | 1 | 19.39101 |
| Dhofar | DJR | 7 | Slope | 3.86 | 849 | 1 | 16.06491 |
| Dhofar | DJR | 8 | Wadi | 7.54 | 836 | 1 | 19.82427 |
| Dhofar | DJR | 9 | Wadi | 9.13 | 831 | 1 | 12.36457 |
| Dhofar | DJR | 10 | Wadi | 5.84 | 831 | 2 | 18.80311 |
| Dhofar | DJR | 11 | Slope | 7.14 | 825 | 1 | 9.908902 |
| Dhofar | DJR | 12 | Slope | 5.78 | 822 | 1 | 13.09422 |
| Dhofar | DJR | 13 | Slope | 4.98 | 823 | 2 | 15.81979 |
| Dhofar | DJR | 14 | Slope | 4.94 | 812 | 1 | 18.71962 |
| Dhofar | DJR | 15 | Slope | 5.22 | 808 | 1 | 13.83822 |
| Dhofar | DJR | 16 | Plateau | 5.64 | 804 | 2 | 10.66099 |
| Dhofar | DJR | 17 | Slope | 5.34 | 803 | 1 | 10.06414 |
| Dhofar | DJR | 18 | Wadi | 6.34 | 801 | 1 | 1.701736 |
| Dhofar | DJR | 19 | Wadi | 5.24 | 801 | 1 | 3.592425 |
| Dhofar | DJR | 20 | Slope | 7.44 | 802 | 2 | 4.70573 |
